# Co-workers' guanxi and construction workers' safety behavior: The mediating role of group identification

**DOI:** 10.3389/fpubh.2022.964514

**Published:** 2022-08-11

**Authors:** Huihua Chen, Wenjing Gong, Hujun Li, Shiying Shi

**Affiliations:** ^1^Department of Engineering Management, School of Civil Engineering, Central South University, Changsha, China; ^2^School of Civil Engineering, Henan Polytechnic University, Jiaozuo, China

**Keywords:** co-workers' guanxi, workers' safety behavior, group identification, mediating role, structural equation model

## Abstract

The construction industry in China is characterized by higher safety risk, and construction workers' unsafe behaviors are one of the main causes of construction safety accidents, thus, designing scientific mechanisms that motivate and cultivate the construction workers to adopt safety behaviors becomes the key to the construction safety problem. Existing studies have examined some of the factors leading to workers' safety behavior (WSB) at the social, organizational, and individual levels, but ignore investigating the impact of co-workers' guanxi (CWG) on WSB. Thus, this research utilized exploratory factor analysis, confirmatory factor analysis, and structural equation modeling to examine the impact of CWG on WSB, and the mediating role of group identification (GI) in the relationship between CWG and WSB. Results show that CWG can directly or indirectly influence WSB, GI can exert a partial mediating effect on the relationship between CWG and GI. The research results enrich the research on c guanxi and causation of WSB, and provide a reference for project managers to carry out relationship-related safety management and industry regulations.

## Introduction

The construction industry is characterized by higher safety risks when compared to other industrial sectors (e.g., manufacturing) (1, 2). Many construction safety accidents across the world happened, which have caused huge tangible and intangible losses to the country, the society, the construction industry, the construction enterprise, and even more serious to the construction workers themselves (3, 4). Therefore, examing the causation of construction safety accidents and then designing proper management strategies to reduce or even avoid these accidents draws more scholars' attention in the academic area (5). The construction workers' safety behavior (WSB) can be widely understood as some construction workers' positive or negative behaviors related to construction safety performance. With delving into the causation of construction safety accidents, many scholars pointed out that the WSB can be directly associated with the construction safety accident, namely workers' unsafe behaviors are the main cause of construction safety accidents (6–8). Hence, designing scientific mechanisms that motivate and cultivate the construction workers to adopt safety behaviors becomes the key to the construction safety problem.

The questions truly triggered many scholars' interests, and the existing literature has identified some antecedents to WSB. These antecedents can be classified into five groups, i.e., individual characteristics, workgroup interactions, work and workplace design, project management and organization, and family, industry and society (6). After analyzing these antecedents in detail, it was found that the existing studies investigated workers' safety behavior based on the western behavioral research paradigm, and ignored that construction workers' behavioral decision-making can't be divorced from the indigenous context of China.The construction worker in China is typically an indigenous working group, in which the basic work unit is mostly a small workgroup (about 7–8 workers included). The small workgroup consists of a foreman (“baogongtou” in Chinese) and some workers associating the foreman with kinship relationships, marital relationships, or fellow-townsman relationships (9, 10). The small workgroup is established based on and maintained by the above-mentioned relationships, or more indigenously, guanxis.

For Chinese construction workers, their long-term life experiences make them believe the above-mentioned innate guanxis as tighter bondages to bring trust rather than relationships derived from contracts and work. Therefore, every construction worker should better maintain guanxi with the foreman and guanxis between every co-worker, and accordingly, these guanxis are critical factors when they conduct behavioral decision-making. Co-workers' guanxi (CWG) is a type of these guanxis existing between every two workers, and it can be kinship guanxi, marital guanxi, or fellow-townsman guanxi. As such, CWG between construction workers might influence their SWBs. However, scholars in construction management still know little about CWG, and also how the concept influence WSB. Group identification (GI) is referred to the extent to which group members recognize and acceptance to their belonging group. A high-level GI can initiate workers to conduct behavior that the group needs. Therefore, when a construction worker has a high-level GI, he (she) may also actively adopt safety behavior. While we also know little about how GI function in the relationship between CWG and WSB. Hence, how does CWG affect WSB? And what role does GI acts in the relationship between CWG and WSB? All of these mentioned questions need more examination.

This study aims to preliminary inquiry on the above-mentioned questions. Firstly, we hypothesized the relationship between CWG and WSG and presumed the mediating role of GI in the relationship between CWG and WSG based on the previous literature. Secondly, we designed a questionnaire and surveyed it to collect data. Thirdly, we carried out an analysis by using the exploratory factor analysis (EFA), confirmatory factor analysis (CFA), and structural equation method (SEM) to validate the conceptual model. The results of the study can provide a reference for management members to take reasonable measures to motivate WSB, help project managers develop safety management strategies related to reasonable guanxi, and provide a basis for regulators to develop industry regulations.

## Literature review and research hypotheses

### Workers' safety behavior

The academic research on WSB can date back to the accident causation theory. Heinrich's domino theory pointed out that workers' unsafe behavior is the primary cause of accidents (11). But at early times, the concept of WSB did not gain more scholars' attention. With prevailing of the philosophy of behavior-based safety, more researchers shift their research interest to WSB. They highlighted that when compared to accident fatality and injury rate, workers' safety behavior can be a more effective predictor of safety performance, and investigating incentive mechanism of safety behavior can provide some ex-ante policies to prevent safety accidents (12).

WSB is not clearly defined by previous scholars, and the basic rule is that the concept or term is “seeing then knowing”. Broadly speaking, WSB can be described as some kinds of behaviors related to direct safety performance (i.e., fatality or injury), thus, workers' safe behavior (positive behavior that can reduce fatality or injury), workers' unsafe behavior (negative behavior that can cause fatality or injury), and safety citizenship behaviors all belong to WSB. However, in a narrow sense, WSB is only associated with some safe behaviors adopted by workers (e.g., wear safety helmets and safety belts) (13). This definition is also widely accepted by area researchers.

In the existing literature, WSB can be interpreted as a one-dimensional concept or a multi-dimensional concept. The first point of view measures the concept as a whole, while the second view argues that WSB should include some components or dimensions. The most classic interpretation is that WSB can be divided into safety compliance behavior and safety participation behavior (14–16). Safety compliance behaviors involve adhering to safety procedures and carrying out work in a safe manner, and safety participation behaviors involve helping coworkers, promoting the safety program within the workplace, demonstrating initiative, and putting effort into improving safety in the workplace (17). Some other scholars offered some different views. For instance, Andriessen (18) divides WSB into attentive behavior and safety active behavior; Larsson et al. (19, 20) classified WSB into structural safety behavior, interactive safety behavior and personal safety behavior; and Gao et al. (21) classified WSB into task-performance safety behaviors and situation-performance safety behaviors.

### Co-workers' guanxi

Guanxi or Guanxi tie is a Chinese indigenous term or phenomenon. This term depicts a state of linkage between people, and the typical guanxi is established based on kinship relations, marital relations and fellow-townsman relations (22, 23). After a long period of social unrest and change, the Chinese have developed their special culture, namely guanxi culture, under the influence of Confucianism (24–30). Influenced by this culture, China's workers try to establish new guanxi and maintain existing guanxi during their social and work transactions (23).

Co-workers' guanxi (CWG) is a typical set of guanxi every worker should maintain when working. The concept can be referred to the relationship between a worker and his (her) workmates. There exist few studies investigating CWG in the academic, but this term can date back to the research on collegiality relationship in business management and pedagogy (31, 32). Collegiality relationship refers to the relationship exiting in colleague (31), and compared to CWG, a collegiality relationship is often established and developed based on formal contracts. However, the research on collegiality relationships in the Chinese context also implied some connotations of CWG.

Collegiality relationship can be a one-dimensional concept or a multi-dimensional concept. As for the multi-dimensional viewpoint, Jing and Yang stated that collegiality relationships can be divided into academic relationships and interpersonal relationships; Li (31) classified collegiality relationships into emotion, tool, obligation and face; Lin et al. (33) validated that collegiality relationship included emotional relationship and instrumental relationship.

### Group identification

Group identification or group identity (GI) is derived from team identification, and team identification can be identified as a continuation of organization identification, which is developed based on social identity theory and self-classification theory (34). Scholars pointed out that in an organization constructed based on work teams, the individual's identification with the team is more effective than his (her) identification with the organization in explaining the individual's attitude and behavior (35), and then team identification becomes the foci of management researchers.

GI refers to the degree to which internal members recognize and accept the group to which they belong (34, 36). The concept is the emotional tendency of group members toward themselves as a group member. This emotional tendency represents the recognition and acceptance of group members to the group they belong to. Most existing literature identified that GI can be divided into three dimensions, i.e., cognitive, evaluative, and affective (34), and validated that GI is an effective predictor of group performance (37–39), individual behavior, individual competence, and psychological characteristics (40–42). As for construction worker, GI can be defined as the degree of recognition and acceptance of the construction group that workers' percept through processes such as self-categorization and affective connection.

### Co-workers' guanxi and workers' safety behaviors

The guanxi literature shows that guanxi can directly or indirectly influence individuals' organizational citizenship behaviors (43–46), extra-role behaviors (47, 48) and even negative behaviors (49–52). Based on the analysis of previous literature it can be concluded that CWG can exert significant influence on individual behavior. Co-worker relationships can directly influence organizational members' behavior, Xu et al. (53) investigated the role of collegiality relationship functioning in the process of organizational innovation, and pointed out that collegiality relationship is a direct influencing variable on employees' innovative behavior. Besides, according to the Theory of planned behavior, action willingness is a proximal antecedent of an individual's certain behavior; when an individual shows strong action willingness, he (she) will adopt that behavior with a high probability (54); and collegiality relationship can affect individuals' action willingness. For example, Li (31) classified collegiality relationships into affective relationships, instrumental relationships, duty relationships, and face, and argued that duty relationships and affective relationships have a positive effect on employees' dedication willingness, while instrumental relationships and face have a negative effect on employees' dedication willingness; Zhang et al. (55) pointed out that the organizational members' social interaction relationship (i.e., collegiality relationship) can positively influence their tacit knowledge sharing willingness.

The completion of every construction workgroup's works requires cooperation among its members. For an individual construction worker, he (she) spent most of his (her) working time with his (her) coworkers, the degree of guanxi with coworkers can influence his (her) safety behavior decision-making. Firstly, based on the analysis of previous paragraph, the collegiality relationship is direct influencing variable of organizational members' behavior, and CWG is derived from the collegiality relationship, thus, CWG can directly influence workers' behavior. Secondly, CWG also involves social norms, favor maintenance, and emotional exchange, and these concepts can affect individual behavior. Therefore, the following hypothesis can be proposed:

H1: There is a positive effect of CWG on WSB.

### The mediating role of group identification

#### Group identification and workers' safety behaviors

A review of GI studies indicates that GI is a significant influencer on individual behavior, and studies have demonstrated that GI can influence employees' discretionary behavior (56), hard-working behavior (41), voice behavior (42) knowledge sharing behavior (36), quality improvement behavior (57), innovation behavior (58, 59), mutual helping behavior (60, 61), and organizational citizenship behaviors (62). The intrinsic reasons are as follow. Firstly, when group members have a strong sense of identification with the group, they will identify themselves with the group as a community of interest. The achievement of individual group goals is based on or included in the process of achieving group goals. Thus, group members will tend to adopt behaviors that are beneficial to the group (such as advocacy behavior and organizational citizenship behavior) (42). Secondly, a higher GI implies a good emotional interaction between group members. Individual has to consider the feelings of other group members when making behavioral decisions, thus, he (she) will adopt specific behaviors to meet the interests of others under certain circumstances (e.g., helping behaviors) (59, 61). Thirdly, action willingness is a significant proximal antecedent of behavior, and GI can also influence group members' action willingness. For instance, Lee et al. (63) showed that GI can influence group members' willingness to attend; Park et al. (64) pointed out that GI has a direct effect on group members' willingness to communicate.

For construction workers, their identification with their affiliated companies and projects is lower due to the project management structure and the current employment status of construction workers in China. Workgroup is the meta-group that carries out construction tasks, workers' identification with the group is more salient. In addition, most construction workers associated with others according to kinship relations, marital relations or fellow-townsman relations, these relations make them easier to cultivate identification. Based on the above-mentioned analysis, the following hypothesis can be proposed:

H2: GI positively affects the WSB.

#### Co-workers' guanxi and group identification

The existing literature identifies that CWG can be an influential variable of GI. Firstly, the exchange between colleagues involves the interchanges of resources, knowledge, and information among members of the same organization (53, 65). These interchanges reflect the organizational members' interdependence, which are prerequisites for organizational members to achieve their own goals. Accordingly, these interchanges lead to organizational members' mutual recognition, and members' sense of identification with their affiliated organization will increase. Secondly, the collegiality relationship involves mutual affection between group members, and this kind of affection will cause mutual concern, mutual understanding, and mutual assistance. Thus, these emotional interactions will help to strengthen employees' identification with the organization (31). Thirdly, the harmonious relationship between employees will also make employees feel “at home” in the organization. This kind of feeling shows the employees' identification with the organization (31). Fourthly, based on the theory of psychological distance, the relationship quality between colleagues characterizes the psychological distance between them. And a closer psychological distance will help develop colleagues' mutual identification which can lead to the incensement of individual group identification (66).

For the group of construction workers, workmates are maintained through interpersonal relationships (guanxi). Based on the above analysis, the collegiality relationship can influence organizational or group identity, and CWG is an indigenous kind of collegiality relationship, thus, it can be inferred that CWG can influence GI. The underlying rule of the inference is the above-mentioned resource exchange and emotion maintenance. Meanwhile, there exist mostly innate ties (i.e., kinship ties, marital ties and fellow-townsman ties) between construction workers. These ties reflect the same culture, upbringing background, and language expressions among construction workers, which can help cultivate mutual identification among the construction workers. Therefore, the authors propose that CWG has an influence on GI and propose the following hypothesis:

H3: CWG positively affects GI.

Based on hypotheses H3 and H2, we can propose the following hypothesis:

H4: GI plays mediating role in the relationship between CWG and WSB.

**Figure 1:**
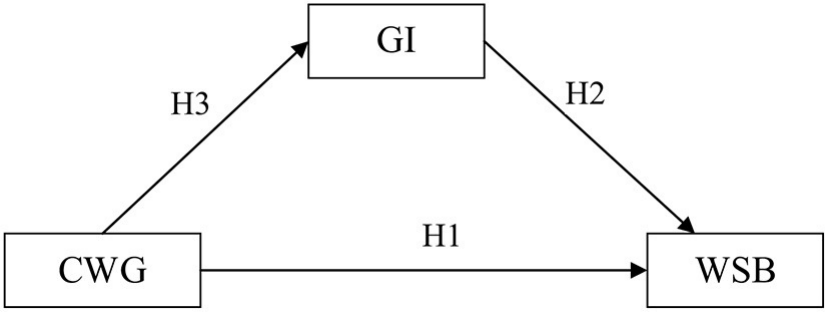
The conceptual model of this study.

Based on the above-listed hypotheses, the conceptual model of this study is shown in Figure 1.

## Research methodology

### Measurement scale design

The measurement scale is the primary component of the questionnaire. Ensuring the validity of the measurement scale can help to gain reliable observation data. We design follow-up steps to ensure the validity of the scale.

Firstly, the questions in the scale were selected from the existing literature. The items to measure CWG were revised based on the scales in Yang's (67) and Chen and Peng's (68) studies; the items in GI scale were selected from Vegt and Stuart's (69) and Smidts' (70) research; the items used to measure WSB were chosen from Xia et al.'s (6), Guo et al.'s (14), and Hu's (71) research.

Secondly, few scholars analyze the CWG in the construction area and there is not a validated scale to measure CWG. We also conducted a semi-structural interview to understand how the construction worker percept CWG. We chose 29 construction workers through personal relationships to conduct the interview. Based on the analysis of the interview text, we revised the CWG scale.

Thirdly, we organized an evaluation team to re-evaluate the scales. The evaluation team includes six researchers and five site experts. All the researchers or experts have ten (or more) years of work or research experience. Based on the team's suggestions, we revised the scales.

Fourthly, the preliminary scales were then sent to six construction workers to do a pre-test. Based on the construction workers' feedback, we further revised the scales to ensure the workers can understand the questions in the scales.

### Procedure and participants

In the questionnaire survey, all the items of co-workers' guanxi, group identification, and workers' safety behavior were measured using a five-point Likert-type response format. Construction workers were asked to endorse the statements using five-point Likert scales (from 1 = strongly disagree to 5 = strongly agree).

We utilized the non-probability sampling technique to select construction workers. After the research team's discussion, Changsha and Zhengzhou were selected as investigating areas, because we have a better cooperation basis with some construction enterprises and construction managers in these areas. This survey strategy ensures we could collect high-quality worker data. Two survey plans were arranged for Changsha and Zhengzhou, respectively. We contacted construction managers, and the construction manager organized frontline workers and distributed the online questionnaire using WeChat. All the surveys were conducted anonymously.

After the two surveys, a total of 278 respondents were received, in which the valid surveys amounted to 271 (the 7 invalid ones were incompletely filled). The sample includes 256 male workers and 15 female workers. They are predominantly aged 20–50. A total of 70 (25.1%) workers were aged 30 or less, 184 (67.8%) workers were aged between 31 and 50, and 19 (7.0 %) workers were aged 51 or over. Respondents' average working time in the construction industry was 9.34 years. The detailed statistical information can be seen in Table 1.

**Table 1:** Demographic information of the participants.

**Measurement items**	**Items options**	**Number of persons**	**Ratio(%)**
Sex	Male	256	94.46
	Female	15	5.54
Education	Elementary school and below	79	29.15
	Junior High School (Secondary)	123	45.39
	High School (Vocational College)	56	20.67
	University and above	13	4.80
Age	≤ 20 years old	4	1.48
	20–30 years old	64	23.62
	31–40 years old	77	28.41
	41–50 years old	107	39.48
	>50 years old	19	7.01
Working years	≤ 1 years	5	1.85
	2–4 years	46	16.97
	5–7 years	74	27.31
	8–10 years	56	20.66
	>10 years	95	35.06

### Data analysis methods

Exploratory factor analysis (EFA), confirmatory factor analysis (CFA) and structural equation modeling (SEM) were applied to conduct the research. EFA was utilized to examine the dimensions of CWG, GI and WSB. Although existing literature has identified GI and WSB could be multi-dimensional concepts. However, a review of WSB and GI research shows that whether the two concepts have dimensions or not changes with the different research. Thus, before we test the concept model, we selected EFA to determine the dimension. CFA was used to examine the validity of the three measure scales. SEM was used to test the conceptual model of this study. The software of SPSS 23.0 and Analysis of Moment Structures (AMOS) 23 was used to conduct EFA and SEM, respectively. The AMOS software is a widely-used software tool for SEM. The significance level was set as 0.05.

## Results

### Data reliability analysis

Cronbach's alpha (CA), Corrected item-total Correlation (CITC), and Cronbach's alpha if item deleted (CA if item deleted) were used to test the reliability of the observed data. The criteria for testing the above three indicators were: the CA was not <0.7; the CITC value was not <0.3; and the CA if an item was deleted could not be greater than the CA of the total measurement scale (72). The reliability of the observed data of this study was tested as follows (can be seen in Table 2).

**Table 2:** Reliability analysis of the observed data.

**Items**	**CITC**	**CA if item deleted**	**Overall CA**
CWG1	0.671	0.685	
CWG2	0.608	0.708	
CWG3	0.539	0.731	
CWG4	0.464	0.756	
CWG5	0.456	0.765	0.772
GI1	0.652	0.725	
GI2	0.594	0.743	
GI3	0.635	0.729	
GI4	0.668	0.717	
GI5	0.303	0.822*	0.791
WSB1	0.416	0.743	
WSB2	0.315	0.762	
WSB3	0.406	0.743	
WSB4	0.432	0.739	
WSB5	0.506	0.727	
WSB6	0.428	0.739	0.779

^*^denotes the item does not pass the test.

For CWG, GI, and WSB, the overall CA was >0.7 and the CITC values were >0.3, indicating that the reliability of the overall measurement scale was high. The CA if item deleted of item GI5 is greater than the overall CA, thus item GI5 should be deleted.

### Exploratory factor analysis

The Kaiser-Meyer-Olkin (KMO) test and Bartlett's test were utilized to examine whether factor analysis can be conducted. The validation criteria are that the KMO values were >0.6, and the *p*-value of Bartlett's test was significant (72). The KMO and Bartlett's test results of CWG, GI and WSB were shown in Table 3.

**Table 3:** KMO and Bartlett's test results of CWG, GI and WSB.

**Concepts**	**KMO test**	**Bartlett's test**
CWG	0.823	*p* ≤ 0.001
GI	0.797	*p* ≤ 0.01
WSB	0.832	*p* ≤ 0.001

Three EFA models were carried out to investigate the dimensions of CWG, GI and WSB, respectively. All the three models show that the CWG, GI and WSB all have one dimension, and the factor loadings of each item can be seen in Table 4.

**Table 4:** Results of EFA and CFA.

**Items**	**Factor loading of EFA**	**Factor loading of CFA**	**Reliability**	**Cronbach's α**	**CV**	**AVE**
CWG1	0.77	0.78	0.61			
CWG2	0.64	0.69	0.48			
CWG3	0.63	0.64	0.41			
CWG4	0.71	0.73	0.53	0.791	0.804	0.507
CWG5	0.47*	0.44*				
GI1		0.74	0.55			
GI2		0.72	0.52			
GI3		0.74	0.55			
GI4		0.76	0.58	0.822	0.829	0.548
WSB1		0.81	0.66			
WSB2		0.58	0.35			
WSB3		0.82	0.67			
WSB4		0.65	0.42			
WSB5		0.78	0.61			
WSB6		0.64	0.41	0.836	0.863	0.512

^*^denotes the item does not pass the test.

### Confirmatory factor analysis

Based on the EFA results, we conducted three CFA for CWG, GI, and WSB. The CFA results were detailed in Table 4. Factor loading, CV, and AVE were utilized to test the validity of the scale. The testing rules of these indices are: factor loading should be >0.5, CV should be >0.7, and AVE should be >0.5 (73).

As can be seen in Table 4, item CWG5 should be deleted according to factor loading rule. After deleting item CWG5, the CWG scale shows better validity (all factor loading of items > 0.5, CV > 0.7, and AVE >0.5). The measurement scale of GI and WSB also show better validity.

### Structural equation analysis and hypotheses testing

**Figure 2:**
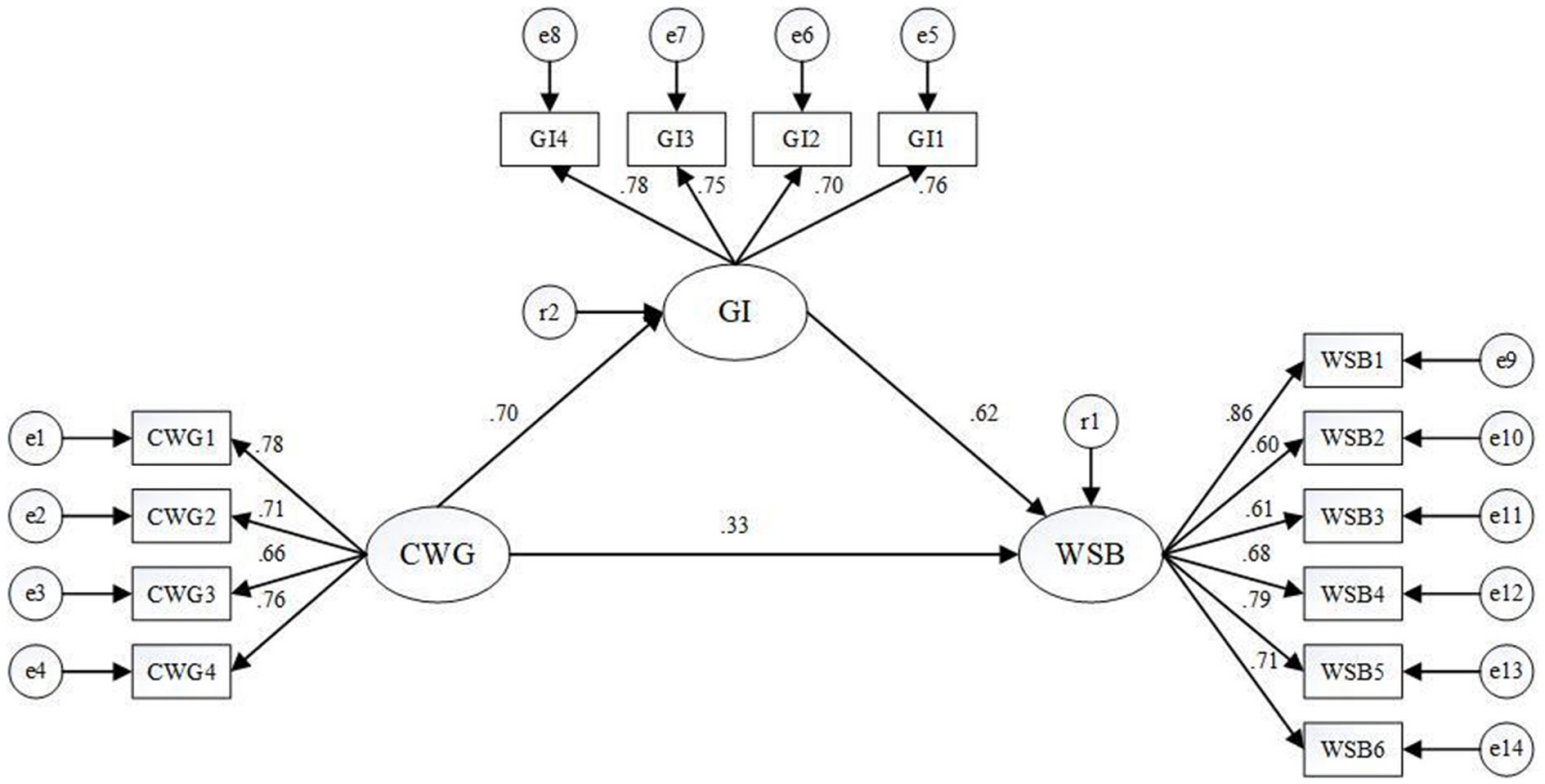
Hypotheses testing results.

The software of AMOS 23 was employed to undertake the SEM analysis for hypotheses testing. The SEM analysis performed to test the conceptual model (shown in Figure 1) revealed an excellent fit (Chi-square/df = 2.603, RMSEA = 0.077, and GFI = 0.901). Figure 2 shows the hypotheses testing results. Solid lines with estimated standardized effect coefficients represent significant links. Besides, we utilized bootstrapping to test the significance of the regression coefficients and mediating effect. After bootstrap 5,000 times, the significance test of the regression coefficients and mediating effect were shown in Tables 5, 6, respectively.

**Table 5:** Significance analysis of regression coefficients.

**Paths**	**Estimates**	**S.E**.	**C.R**.	**Bootstrap 5000**				**P**
				**Bias-corrected**		**Percentile**		
				**Lower**	**Upper**	**Lower**	**Upper**	
GI < -CWG	0.758	0.109	6.177	0.488	0.856	0.505	0.881	***
WSB < -CWG	0.357	0.078	4.456	0.158	0.536	0.162	0.557	***
CSB < -GI	0.671	0.085	6.238	0.367	0.763	0.361	0.753	***

*** refers to p-values less than 0.01.

**Table 6:** Significance analysis of the mediation effect.

**Paths**	**Point estimate**	**S.E**.	**Z**	**Bootstrap 5000**				**P**
				**Bias-corrected**		**Percentile**		
				**Lower**	**Upper**	**Lower**	**Upper**	
Indirect effect	0.434	0.117	3.71	0.229	0.637	0.231	0.547	***
Direct effect	0.325	0.104	3.13	0.123	0.489	0.221	0.504	***
Total effect	0.759	0.212	3.58	0.602	0.921	0.623	0.948	***

*** denotes that p-values < 0.01.

Hypothesis 1 proposed that CWG can exert a positive effect on WSB. As can be seen in Figure 2 (path coefficient = 0.33), Table 5 (estimates = 0.357) and Table 6 (direct effect = 0.325), the regression coefficient and direct effect are both significant (*p* < 0.01), which demonstrates that the hypothesis 1 was validated.

Hypothesis 2 proposed that GI can exert a positive effect on WSB. As can be seen in Figure 2 (path coefficient = 0.62) and Table 5 (estimates = 0.671), the regression coefficient is significant (*p* < 0.01), which demonstrates that the hypothesis 2 was validated.

Hypothesis 3 proposed that CWG can exert a positive effect on GI. As can be seen in Figure 2 (path coefficient = 0.70) and Table 5 (estimates = 0.758), the regression coefficient is significant (*p* < 0.01), which demonstrates that the hypothesis 3 was validated.

Hypothesis 4 proposed that GI plays mediating role in the relationship between CWG and WSB. As can be seen in Table 6 (indirect effect = 0.434), the direct effect is significant (*p* < 0.01), which demonstrates that GI functions as a positive mediator in the relationship between CWG and WSB. And because Hypothesis 1 was also validated, it can be concluded that GI partially exerts a positive mediating effect on the relationship between CWG and WSB. Besides, according to Table 6, the total positive effect CWG exerts on WSB is 0.759, which implies CWG is a significant predictor of WSB.

## Discussion

### Impact of co-workers' guanxi on workers' safety behavior

This study validates that CWG can exert a positive direct and indirect effect on WSB, which is consistent with previous literature. Firstly, CWG can be derived from collegiality relationship, previous studies have shown that collegiality relationship can directly or indirectly influence group members' innovative behavior and action willingness (52, 54). Besides, for construction workers, CWG quality reflects whether the two parties can harmoniously cooperate on the construction site, and the behavior of co-workers toward safety can directly or indirectly affect the workers' behavior, namely, the behavior propagation mechanism (9, 74). This study reveals that CWG is a salient predictor of WSB. This findings have some theoretical implications. Firstly, the study enrich the knowledge of the WSB causation theory and the indigenous guanxi theory. Secondly, this study provides a new perspective to investigate the motivation of WSB by introducing CWG into construction.

### Mediating effects of group identification

GI was shown to have a mediating effect on the process of CWG influencing WSB. Based on the review of previous studies, it is clear that the mediating effect of GI is reasonable. Firstly, CWG originates from collegiality relationship. Previous studies have pointed out that collegiality relationship involves resource exchange, information exchange, emotions exchange, and closeness among organization members. These concepts all contribute to employees' mutual identification (65, 75, 76). Secondly, GI is derived from organizational identification, and organizational identification directly influences organizational voice behavior, innovation behavior, mutual support behavior (77) and organizational citizenship behaviors (78), etc. This study reveals that GI is a mediating variable in the relationship between CWG and WSB, which deepens previous guanxi-behavior research, and introduces the research paradigm of “guanxi-identity-behavior” to the research upon construction workers' group. This study reveals that GI can exert a positively mediating effect on the relationship between CWG and WSB. Although this research is preliminary, it explains that “the black box”, namely the relationship between CWG and WSB is complex. This finding can not only enrich the knowledge of the WSB causation theory, but also provide new research agenda. Follow-up research can further examine the role of other concept (e.g., knowledge-sharing, team cooperation) in the relationship between CWG and WSB.

## Management implication

The study validated CWG was an important influencer to WBS. Attention should be paid to the cultivation of CWG, and when CWG quality is higher, construction managers can effectively carry out WSB supervision. The construction group managers can cultivate high-quality CWG from the following ideas: (1) enhance personal relationships at the group level by organizing group recreational activities during non-working hours; (2) reduce punitive measures on workers' unsafe behavior and enhance the role of guanxi supervision. The implementation of punitive measures for unsafe behavior can seriously harm workers' feelings and make them rebellious. In contrast, relationship supervision emphasizes the emotion interaction among construction workers, which can make them agree and understand each other, and then strengthen construction workers' willingness to adopt safe behaviors.

GI can direct influence WSB. For construction managers, they can identify and design the common features of the group from the following ideas: (1) promote communication using dialect within the whole group; (2) unify the work clothes of the group members; (3) actively cultivate common hobbies, and (4) design a unified work slogan. Besides, it is possible to enhance construction workers' emotional identification by organizing collective recreational activities and experience-sharing meetings.

## Limitations

This study suggests that CWG can exert a direct and indirect effect on WSB, and GI has positively mediating effects on the relationship between CWG and WSB. However, the research reported in this paper does have some limitations. Firstly, the study is a preliminary investigation of CWG, thus the measurement scale might be less perfect. Research on guanxi and collegiality relationship identified relationship or guanxi might be a multi-dimensional concept. Follow-up researchers can pay attention to the measurement of CWG. Secondly, this research examined the function of GI in the relationship between CWG and WSB, however, there might be some other mediating variables (e.g., group knowledge sharing, individual culture). Therefore, researchers can further investigate the mechanism of how CWG influencing WSB. Thirdly, the research reported in this paper was undertaken in Changsha and Zhengzhou. It is encouraged that similar studies can be undertaken in other China areas to further validate the rationality of the theory proposed in this paper.

## Conclusion

This study employs exploratory factor analysis, confirmatory factor analysis, and structural equation modeling to examine the impact of CWG on WSB, and the mediating role of GI in the relationship between CWG and WSB. Following conclusion can be reached based on above analyses.

(1) CWG can exert a directly positive effect on GI and WSB, and GI can positive influence WSB.
(2) CWG can also exert an indirectly positive on WSB, of which GI functions as a partial mediating variable.

## Data availability statement

The original contributions presented in the study are included in the article/Supplementary material, further inquiries can be directed to the corresponding author/s.

## Ethics statement

Ethical review and approval was not required for the study of human participants in accordance with the local legislation and institutional requirements. Written informed consent from the participants was not required to participate in this study in accordance with the national legislation and the institutional requirements.

## Author contributions

HC plays a guiding role in this study. WG is responsible for the writing. HL is in charge of research and revision. SS is responsible for data processing. All authors contributed to the article and approved the submitted version.

## Funding

This work was supported by the Special Project of National Natural Science Foundation of China (71942006), Sichuan Provincial Science and Technology Program Project (2020JDR0396), and Doctoral project of Henan Polytechnic University (B2022-23).

## Conflict of interest

The authors declare that the research was conducted in the absence of any commercial or financial relationships that could be construed as a potential conflict of interest.

## Publisher's note

All claims expressed in this article are solely those of the authors and do not necessarily represent those of their affiliated organizations, or those of the publisher, the editors and the reviewers. Any product that may be evaluated in this article, or claim that may be made by its manufacturer, is not guaranteed or endorsed by the publisher.
