# Supplementary material for: Co-workers' guanxi and construction workers' safety behavior: The mediating role of group identification
Source: Front Public Health. 2022 Aug 11;10:964514. doi: 10.3389/fpubh.2022.964514 (PMC9403466; doi:10.3389/fpubh.2022.964514)
Supplement: Supplementary file 1 [file Table_1.pdf]

Appendix 1. Measurement Scale

Co-worker guanxi Measurement Scale					
Measurement question items	Strongly agree	Somewh at agree	Generall y agree	Not quite agree	Disa gree
Helping each other is the rule of work between me and my workmates					
I share my views with my workmates and respect each other's views					
I often go to dinner, drink, shopping and sports with my workmates					
I will take into account the thoughts of the workers when making decisions					
My workmates are trustworthy					
Group identification Measurement Scale					
Measurement question items	Strongly agree	Somewh at agree	Generall y agree	Not quite agree	Disa gree
Feel an emotional attachment to our group					
Feel a strong sense of belonging to our group					
It feels like the group's problem is our problem					
Feels like home in the group					
It makes sense for the foreman or senior to ask me to do something					
Workers' safety behavior Measurement Scale					
Measurement question items	Strongly agree	Somewh at agree	Generall y agree	Not quite agree	Disa gree
I strictly follow the project safety management norms to wear safety protective equipment, such as helmets, masks, etc.					
I carry out construction operations in strict accordance with safety management codes and manuals					
I conduct a rigorous inspection of safety equipment before operation					
I will help the workers to ensure they work safely					

I will actively participate in the project's safety education training					
I will actively do more work to ensure site safety					
